# Molecular mechanism of somatic embryogenesis in *paeonia ostii* ‘Fengdan’ based on transcriptome analysis combined histomorphological observation and metabolite determination

**DOI:** 10.1186/s12864-023-09730-6

**Published:** 2023-11-03

**Authors:** Wanqing Zhang, Hongxiao Zhang, Guodong Zhao, Na Wang, Lili Guo, Xiaogai Hou

**Affiliations:** 1 Agricultural college, Henan University of Science and Technology, 471023 Luoyang, Henan China; 2 National Peony Gene Bank, 471011 Luoyang, Henan China

**Keywords:** Tree peony, Embryogenic callus, Somatic embryo, Histomorphology, Transcriptome

## Abstract

**Background:**

Tree peony (*Paeonia* sect. *Moutan* DC.) is a famous flower native to China with high ornamental, medicinal, and oil value. However, the low regeneration rate of callus is one of the main constraints for the establishment of a genetic transformation system in tree peony. By histomorphological observation, transcriptomic analysis and metabolite determination, we investigated the molecular mechanism of somatic embryogenesis after the establishment of a culture system and the induction of somatic embryo(SE) formation.

**Results:**

We found that SE formation was successfully induced when cotyledons were used as explants. A total of 3185 differentially expressed genes were screened by comparative transcriptomic analysis of embryogenic callus (EC), SE, and non-embryogenic callus (NEC). Compared to NEC, the auxin synthesis-related genes *GH3.6* and *PCO2* were up-regulated, whereas cytokinin dehydrogenase (*CKX6*) and CYP450 family genes were down-regulated in somatic embryogenesis. In SE, the auxin content was significantly higher than the cytokinin content. The methyltransferase-related gene S-adenosylmethionine synthase (*SAMS*) and the flavonoid biosynthesis-related gene (*ANS* and *F3’5’H*) were down-regulated in somatic embryogenesis. The determination of flavonoids showed that rhoifolin and hyperoside had the highest content in SE. The results of transcriptome analysis were consistent with the relative expression of 8 candidate genes by quantitative polymerase chain reaction analysis.

**Conclusion:**

The results revealed that auxin and cytokinin may play a key role in ‘Fengdan’ somatic embryogenesis. The genes related to somatic embryogenesis were revealed, which has partly elucidated the molecular mechanism of somatic embryogenesis in ‘Fengdan’.

**Supplementary Information:**

The online version contains supplementary material available at 10.1186/s12864-023-09730-6.

## Background

Tree peony (*Paeonia* sect. *Moutan* DC.) is a famous flower native to China with high ornamental, medicinal and oil value [1, 2]. *Paeonia ostii* ‘Fengdan’ is a cultivated species of tree peony and one of the emerging woody oil plants in China because of its high α-linolenic acid content in seeds [1, 3]. In recent years, although the planting area of ‘Fengdan’ has rapidly increased, the conventional methods of reproduction have been unable to meet the market demands due to the long period required for reproduction [4]. Therefore, research involving peony tissue culture is valuable and may provide the basis for its suspension cell and seedling culture. It is also important for molecular breeding [5]. Generally, the establishment of a callus regeneration system includes four stages, namely callus induction, embryogenic callus domestication, somatic embryos formation, and regenerated plantlets development. Induction of the embryogenic callus is the key step for the rapid acquisition of regenerative plants [6]. Ganesan et al. reported that different callus morphologies have different effects on its differentiation, and the metabolic ability, as well as the meristem ability, embryogenic callus were at a high level [7]. A previous study reported that SE can be successfully induced from zygotic embryos of *Paeonia rockii* and ‘Fengdan’ [6]. In addition, in a study of 6-BA and NAA treatment in ‘Fengdan’, it was found that zygotic embryos and cotyledons can effectively produce SE. [8]. At present, reports of the molecular mechanism of somatic embryogenesis in ‘Fengdan’ are lacking.

The developmental transition is triggered at the callus induction stage, which determines the factors for calli to transform into SE and regenerated plants. These factors include differentially expressed genes (DEGs) and various signal transduction pathways that can activate or inhibit multiple genes [9]. However, the regenerative efficiency of ‘Fengdan’ callus is extremely low, which constraints the application and transformation of transgenic technology in trait improvement and breeding [10].It was demonstrated that indole-3-acetic acid (*IPA*), auxin (*AUX*) and isopentenyl transferase (*IPT*) are up-regulated in embryonic callus during cucumber cotyledon node differentiation [11]. In maize study, *ZmBBM2* overexpression can promote the induction and proliferation of callus [12]. In *Eucalyptus*, several somatic embryogenesis related genes, namely17 ethylene genes, 12 auxin genes, 122 cell wall-related genes, and 98 transcription factors, were identified [13]. Exogenous supplementation with epiBR promotes the embryogenesis of callus and the expression of somatic embryos and auxin-related genes. Sandra Correia investigated the role of the rRNA methyltransferase NEP-TC on somatic embryogenesis in Solanum betaceum Cav. and found that when NEP-TC was down-regulated, the induction level of somatic embryos was slightly increased [14]. In addition, recent advancements in transgenic technology are providing new insights on the regulation of callus formation [15]. The calcium (Ca^2+^) signaling module CaM-IQM can interacts with auxin to regulate callus and lateral roots formation [16]. The chimeric *GRF4-GIF1* gene is required for efficient transformation in wheat, and dicotyledonous *GRF-GIF* chimeras showed improved efficiency and regeneration [17]. At present, several molecular techniques are available to analyze the regulatory networks at different stages of plant development. In tissue culture, overexpression of plant development regulatory factors improves plant regenerative efficiency. However, proliferating embryogenic callus often loses its ability to regenerate due to epigenetic variation. DNA methylation and *H3K27me3* demethylation promote callus development in an epigenetic study of the transformation of peach leaves into callus [18]. In addition, the low differentiation rate of ‘Fengdan’ embryonic callus is due to hypermethylation by the methylation sensitive amplification polymorphism technique [19]. RNA-sequencing (RNA-seq) is widely used in the identification of key genes that regulate plant growth and development as well as the construction of high-density genetic maps [5, 13, 20]. Therefore, in this study, RNA-seq was used to analyze the different developmental stages of ‘Fengdan’ callus, and the key genes involved in somatic embryogenesis were determined to understand the molecular mechanism of somatic embryogenesis in ‘Fengdan’.

## Materials and methods

### Callus culture and somatic embryo induction

‘Fengdan’ seeds were obtained from the National Peony Gene Bank, Luoyang, China. Sized full and glossy seeds were selected for the experiment. Seed coats were removed, and seeds were soaked in sterilized water for 24 h and then sterilized on an ultra-clean work table. Embryos were cut and inoculated into Murashige and Skoog (MS) medium, containing 0.8 mg/L NAA, 1.0 mg/L 6-BA and 1.0 g/L activated charcoal for aseptic seedling culture. ‘Fengdan’ hypocotyls and cotyledons were selected as explants. Three basic media including MS medium, vitamin B5 was added to MS medium (MB) and sucrose was replaced with glucose (MBP). Using the three kinds of culture medium, and 2,4-dichlorophenoxyacetic acid (2,4-D), thidiazuron (TDZ), 6-benzylaminopurine (6-BA), and 1-naphthaleneacetic acid (NAA) were used to prepare 9 treatments (Table 1) for callus to induction and SE formation. The somatic embryo induction culture was carried out in light conditions at a temperature of 25 °C after 7 d of culture in dark conditions. One culture cycle was 30 d. For each treatment, 30 bottles were inoculated, and each bottle was inoculated with 3–6 explants. By stereomicroscopy non-embryogenic callus (NEC) and embryogenic callus (EC) cultured for 25 d were selected, and successive generation of SE for 15 d was used for histomorphological observation, transcriptome sequencing, and hormone detection. Nine replicates for each sample were taken; three replicates were immersed in formaldehyde-acetic acid-ethanol (FAA) fixative for 24 h and stored at 4 °C, and the other six replicates were immediately frozen in liquid nitrogen, and stored at -80 °C until use.

**Table 1:** Induction of SE Formation by Different Treatments

Treatment	Medium	2,4-D (mg/L)	TDZ (mg/L)	NAA (mg/L)	6-BA (mg/L)	SE from Cotyledon (%)	SE from Hypocotyl (%)
S1	MBP	0.5	0	1.0	0	3.75 ± 2.67c	16.62 ± 7.74ab
S2	MS(Ca^2+^)	0.5	0.25	0	0	28.67 ± 3.45ab	29.99 ± 7.37a
S3	MS(Ca^2+^)	0.5	0	1.0	0	3.33 ± 2.22c	16.67 ± 7.03ab
S4	MS(Ca^2+^)	0	0	1.0	0.5	6.67 ± 4.44c	0.00 ± 0.00c
S5	MS(Ca^2+^)	0.5	0	0	0.2	19.33 ± 7.24b	5.33 ± 2.73bc
S6	MB	0.5	0	0	0.2	0.00 ± 0.00c	0.00 ± 0.00c
S7	MBP	0.5	0.25	0	0	31.61 ± 3.31a	12.58 ± 4.12bc
S8	MBP	0	0	0.5	0.2	0.00 ± 0.00c	0.00 ± 0.00c
S9	MS(Ca^2+^)	0	0	0.5	0.2	0.00 ± 0.00c	6.67 ± 4.44bc

Note: MBP: MS basal medium supplemented with vitamin B5 and glucose instead of sucrose, MS (Ca^2+^): Double Calcium in MS Medium, MB: MS basal medium supplemented with vitamin B5. SE: Somatic embryo. 6.8 g/L agar and 30 g/L sucrose were added to each medium. Cotyledon somatic embryo formation rate: Somatic embryo formation rate when cotyledons were as explants. Hypocotyl somatic cell embryo formation rate: Somatic embryo formation rate when hypocotyl as explants. Somatic embryo formation rate= (Number of explants forming somatic cell embryos/ Number of inoculated explants)×100%. Data are the means ± standard error of three replicates. The difference in the mean of different letters in the same column was statistically significant (*P* < 0.05). Values are means ± SE (n = 3) of three different experiments. Means denoted by letters refer to the significant differences (*P* < 0.05, Duncan’s test)

### Histomorphological observation

The different stages of somatic embryogenesis were photographed with Nikon MacroShot and 3D Zeiss microscopes, and histological observation was performed. Before treatment, the materials were subjected to vacuum pumping for 30 min, followed by immersion in FAA fixative at room temperature for more than 24 h, gradient dehydration with ethanol, and embedment with paraffin. Using the Leica RM2235 microtome, the tissue blocks were cut to generate 8–10 μm thick sections, and histological observation was performed after hematoxylin staining.

### RNA-seq, cDNA library construction, and transcriptomic analysis

A total of nine samples from the three groups were sent to Beijing Biomarker Biotechnology Company (https://international.biocloud.net/zh/dashboard). The samples provided included NEC, EC, and SE. RNA was extracted using a polysaccharide polyphenol total RNA kit (Tiangen, Beijing, China), and the RNA quality was determined by NanoDrop spectrophotometer. The cDNA library was constructed according to Illumina instructions. The cDNA library volume was ≥ 10 µl, and the concentration was ≥ 40 ng/µl. The double-stranded cDNA of the 9 samples were sequenced on the Illumina platform of Beijing Biomarker Biotechnology Company, Beijing, China. The raw transcriptome sequencing data were stored in the National Center for Biotechnology Information (NCBI) database (accession no. PRJNA901227).

A large number of high-quality raw data were generated through the Illumina high-throughput platform. High-quality clean reads were obtained by FastQC (Babraham Institute, Cambridge, UK) filtering. Each sample had two FASTQ files containing cDNA reads measured at both ends. Adapter sequences containing nucleotides with low quality-scores were removed. Data processed by the above steps were designated as clean data (≥ Q30%). Clean data were provided in FASTQ format. Comparisons were made with the tree peony genome sequence (https://db.cngb.org/search/project/CNP0000281/). Paired terminal clean reads were compared with the reference genome using HISAT2v2.1.0 (Center for Computational Biology, USA). The alignment efficiency of the clean reads of each sample with the specified reference genome ranged from 73.52% to 76.41%. After splicing mapped reads with StringTie v2.1.5 (Center for Computational Biology, USA) software, the full-length cds sequence and annotation information of each gene were obtained, and new genes were diacovered. StringTie was used to statistically align read count values to each gene as a base. The original expression quantity of fragments per kilo basesper million was used to normalize the expression levels [21]. The BLAST software was used to compare the new genes with various databases, including NR, Swiss-Prot, Pfam, KEGG, KOG, COG, KO, and GO.

### Analysis of differentially expressed genes and functional annotation

DESeq was used to compare the single genes between samples, and log_2_ (Fold Change, FC) ≥ 1, *P-*value < 0.05, and false discovery rate (FDR) < 0.001 were used as screening criteria for the selection of the DEGs. GO and KEGG [22] functional annotation of DEGs was performed by Biomarker cloud platform.

### Validation of DEGs using qRT-PCR

The expression of EC and SE related DEGs was detected by qRT-PCR (Bio-Rad CFX96, USA). RNA was extracted using a polysaccharide polyphenol total RNA kit (Tiangen, Beijing, China). Ten µl of RNA was extracted from each sample. The quality and quantity of RNA were assessed by 1.2% agarose electrophoresis and NanoDrop 2000c spectrophotometry (Thermo Scientific, USA) respectively. The reverse transcribed with AG *Evo M*-*MLV* RT Mix Reverse Transcription Kit, removes DNA residues from the genome with gDNA Clean Reaction Mix Ver.2 and Fluorescence quantitative PCR was using the SYBR® Green *Pro Taq* HS Master Mixed qRT-PCR Kit. The reaction was comprised of 1 µl of cDNA template, 10 µl of 2 × SYBR Green Pro Taq HS premix, 0.4 µl each of primer F and primer R (10 µmol·L^-1^), and RNase-free H_2_O to 20 µl. The cycling conditions were as follows: pre-denaturation at 95 °C for 30 s, followed by 40 cycles of denaturation at 95 °C for 5 s and renaturation at 60 °C for 30 s. Each sample was tested 3 times. Normalization was performed using the *EF1-α* gene as the internal control [23]. Data were analyzed by Microsoft Excel 2019 software, and the relative expression was calculated by the 2^-ΔΔCt^ method [24]. The primers were designed using Primer-BLAST, and they are listed in Table S1.

### Hormone determination

In this experiment, NEC and SE were used as the materials. The AGLIENT1260 high performance liquid chromatography tandem 6420 A mass spectrometry (AGLIENT Company, US) was used for the determination of hormone content. The ionization mode of the mass spectrometer was ESI positive and negative ion modes, and the scanning type was multiple reaction monitoring (MRM). The curtain gas of the mass spectrometer was 15 psi, the spray voltage was + 4500 v and − 4000 v, the atomization gas pressure was 65 psi, the auxiliary gas pressure was 70 psi, and the atomization temperature was 400 °C. Methanol solution was used to prepare Indole-3-aceticacid (IAA), N6-isopentenyladenine (IP), indolepro pionic acid (IPA), zeatin, and the standard curve were generated. The samples (0.5 g) were ground into a powder in test tubes with liquid nitrogen. Extraction was performed overnight at 4 °C with 10 times the volume of acetonitrile solution,and the sample was centrifuged at 12,000 rpm for 5 min to absorb the supernatant. A 120 SB-C18 reversed-phase chromatographic column (2.1 × 150, 2.7 μm) was used for the determination. The mobile phases were 0.1% formic acid solution in water (solvent A) and acetonitrile - methanol mixture (solvent B). The elution gradient was 20% for 0–1 min, 20% increase to 50% for 1–3 min, 50% increase to 80% for 3–9 min, 80% for 9–10.5 min, 80% for 10.5–10.6 min, 20% for 10.6–13.5 min. The injection volume was 2 µl, and the flow rate was 0.3 ml/min at 30℃. The raw data files of hormone determination were deposited at Figshare (https://figshare.com/s/e1f1bbdc5f03fbb7c647). The hormone content was determined using the following formula:

Hormone content (ng/g) = detection concentration (ng/ml) × dilution volume (ml) / weight (g).

### Flavonoid determination

In this experiment, NEC, SE and somatic embryos induced by 0.6 mg/L methylation inhibitors (5-azacytidine) for 30 d were used as the materials. The determination method was the same as that for plant hormones. 40 kinds of flavonoid standard solutions were prepared using methanol solution. The samples (1.0 g) were ground to powder in test tubes with liquid nitrogen, and 1% hydrochloric acid–methanol solution was added, followed by sonication in an ice bath for 30 min. The samples were centrifuged at 11,000 rpm for 30 min. The supernatant was filtered through 200 μm organic phase filters and stored at -20 °C. The chromatographic column was Agilent ZORBAX Eclipse Plus C18 (3.5 μm, 2.1 × 150 mm), and the mobile phases were 0.3% formic acid solution in water (solvent A) and 0.3% formic acid solution in acetonitrile (solvent B). Gradient elution was performed, and the flow rate was 0.3 ml/min. The raw data files for the determination of flavonoids was deposited at Figshare (https://figshare.com/s/e1f1bbdc5f03fbb7c647). Flavonoid content was calculated by the same method as the hormone content.

### Statistics analysis

The statistical indicators were expressed as the average of 3 replicates. One-way analysis of variance was used, and Duncan’s range test was performed. Histological observations were performed at least five times with similar results. Different letters were used to indicate significant differences (*P* < 0.05, Duncan’s test). Statistical analysis was performed using SPSS v25 (IBM,Armonk, NY, USA), and Origin 2018 (Northampton, Massachusetts, USA) was used to make the histogram.

## Results

### Somatic embryo induction and morphological and histological observations

**Fig. 1:**
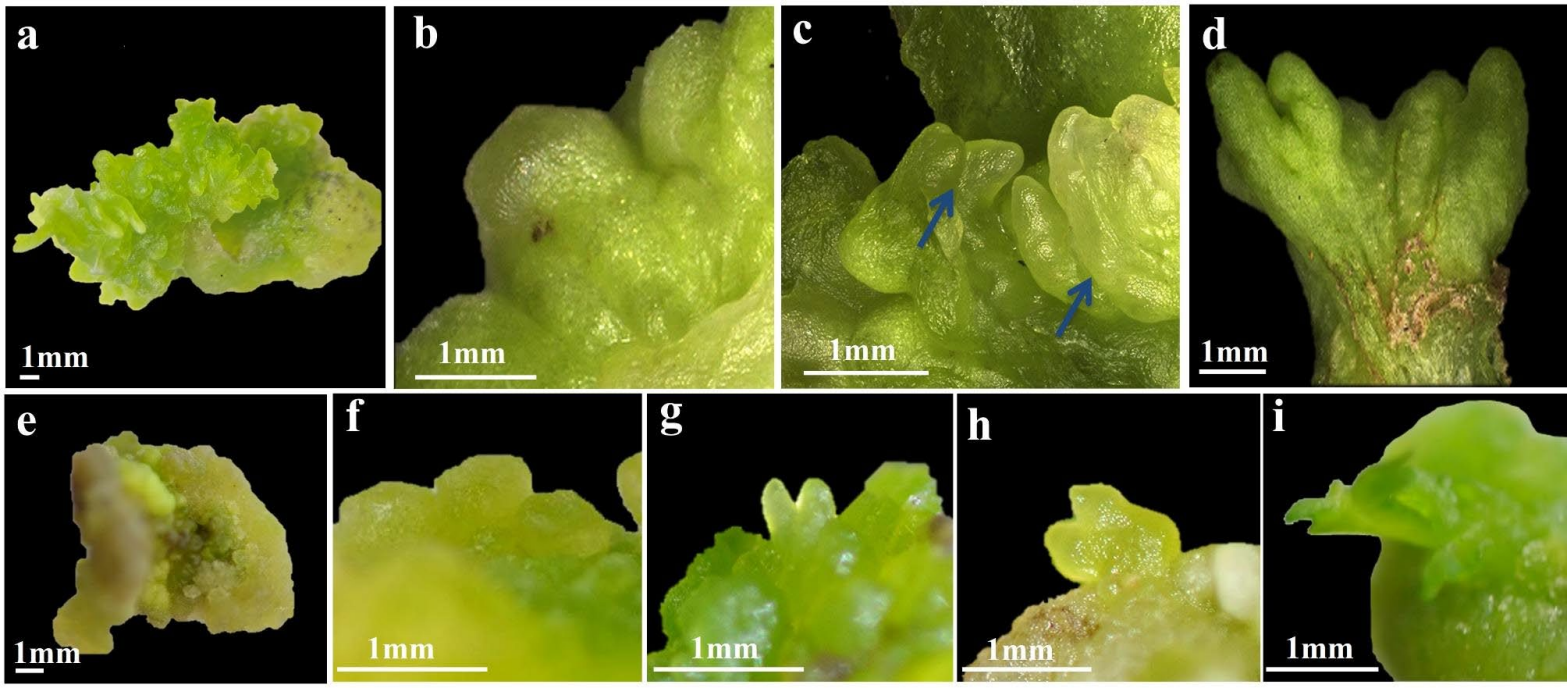
Observations on apparent morphology of ‘Fengdan’ somatic cell embryos at different developmental stages. **(a-d)** Direct induction of somatic embryo formation by cotyledon. **(a)** Callus was induced for 25 d in the primary generation when the cotyledon was the explant, **(b)** Formation of spherical embryos on callus surface at 25 d of primary culture, **(c)** After 15 d of subculture, heart-shaped embryos, and torpedo embryos were formed on the callus surface, **(d)** Formation of cotyledon embryos on the callus surface after 25 d of subculture. **(e-i)** Indirect somatic embryo formation induced by hypocotyls, **(e)** Callus was induced for 45 d when the hypocotyl was an explant, **(f)** Spherical embryo of callus subcultured for 15–20 d, **(g)** Calli were subcultured to form heart-shaped embryos at 35 d, **(h)** Callus was subcultured for 40 d to form torpedo embryos, **(i)** The callus was subcultured for 45 d to form cotyledon embryos

MS, MB and MBP were used as the basic medium for induction. To investigate the effects of hormones on SE differentiation, a total of 9 groups of medium combinations were set up (Table 1). Cotyledons were used as explants, and SE was directly formed on MBP based medium (Fig. 1a). The highest induction rate was 31.61 ± 3.31%, when 0.5 mg/L 2,4-D combined with 0.25 mg/L TDZ. When hypocotyls were used as explants, SE could only be differentiated through EC formation, which was considered indirect SE formation (Fig. 1e-i). The optimum medium ratio was MS + 0.5 mg/L 2,4-D + 0.25 mg/L TDZ. Through the observation of callus induction of ‘Fengdan’, it was found that cotyledons could directly induce the formation of SE, and spherical embryos on the surface of callus gradually differentiated into heart-shaped embryos, torpedo embryos, and cotyledon embryos (Fig. 1b-d).

**Fig. 2:**
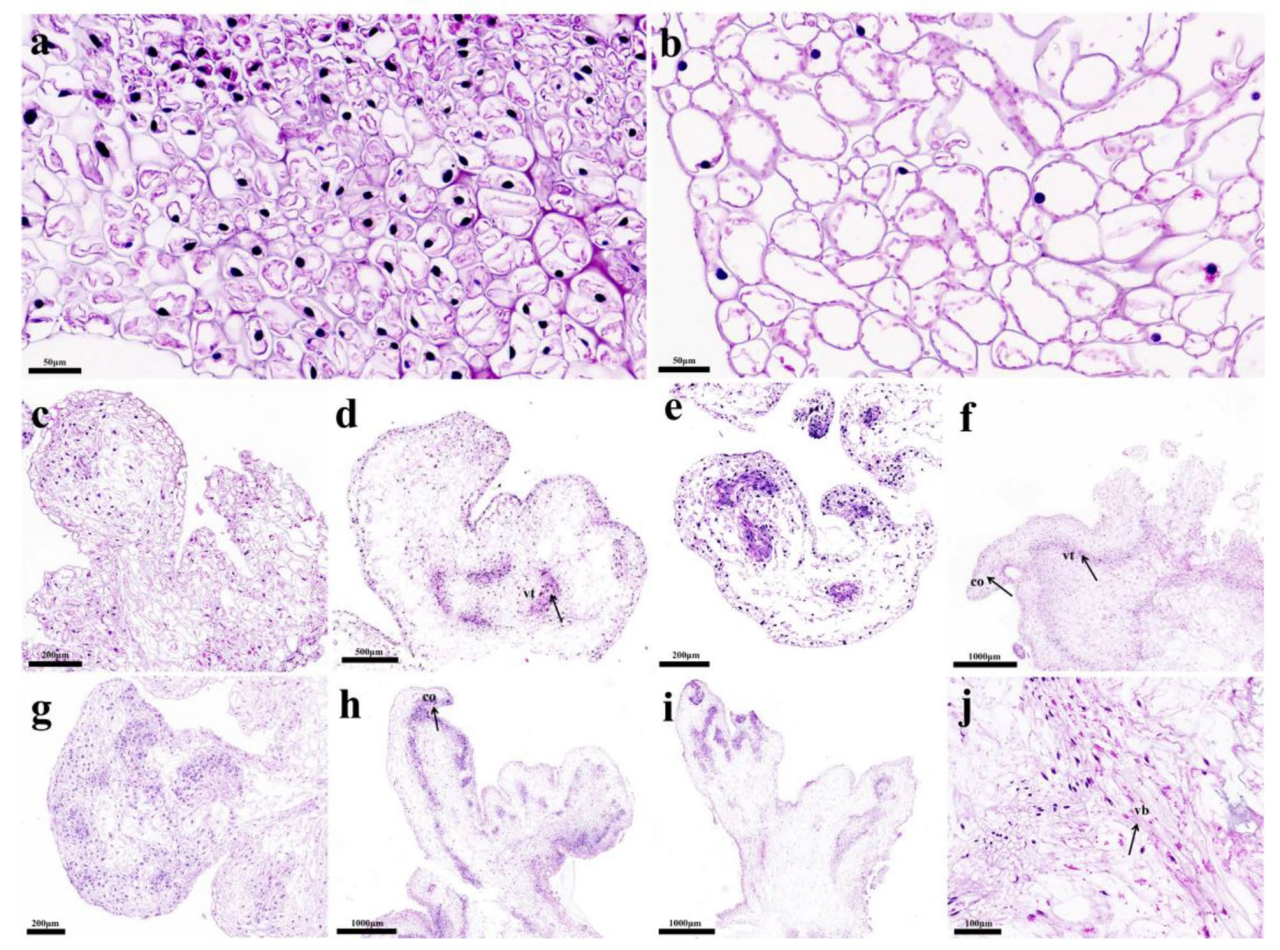
Histological observation of ‘Fengdan’ somatic cell embryos at different developmental stages. **(a)** Embryonic cells, **(b)** Non-embryonic cells, **(c)** Spherical embryo cells formed by indirect somatic embryos, **(d)** Heart-shaped embryo cells formed by indirect somatic embryos, **(e)** Torpedo embryo cells formed by indirect somatic embryos, **(f)** Cotyledon embryo cells formed by indirect somatic embryos, **(g)** Spherical embryo cells formed by direct somatic embryos, **(h)** Torpedo embryo cells formed by direct somatic embryos, **(i)** Cotyledonary embryo cells fromed by direct somatic embryos, **(j)** Local amplification of vascular tissue. vt: vascular tissue, co: cotyledon, vb: vascular bundle. Scale: a,b = 50 μm, j = 100 μm, c,e,g = 200 μm, d = 500 μm, f,h,i = 1000 μm

SE at different developmental stages of ‘Fengdan’ were histologically observed using paraffin sections. It was found that the nucleus of EC was larger, and the cytoplasm was thicker (Fig. 2a), whereas the nucleus of NEC was less and the cytoplasm was thin. (Fig. 2b). As the EC continued to differentiate, they gradually formed spherical embryos with distinct globular structures (Fig. 2c, g). With the development of SE, cell differentiation at both ends of the globular embryos was accelerated, forming heart-shaped embryos and torpedo embryos (Fig. 2d,e,h). A large number of starch granules accumulated in torpedo embryo and began to form vascular tissues. Cotyledon shaped embryos with shoot and stem meristem were formed by subculture (Fig. 2f, i). During the process from globular embryos to cotyledon embryos, SE gradually differentiated from maternal tissues to vascular bundles (Fig. 2j), which indicated the reliability of SE formed in this study.

### Transcriptomic analysis

**Fig. 3:**
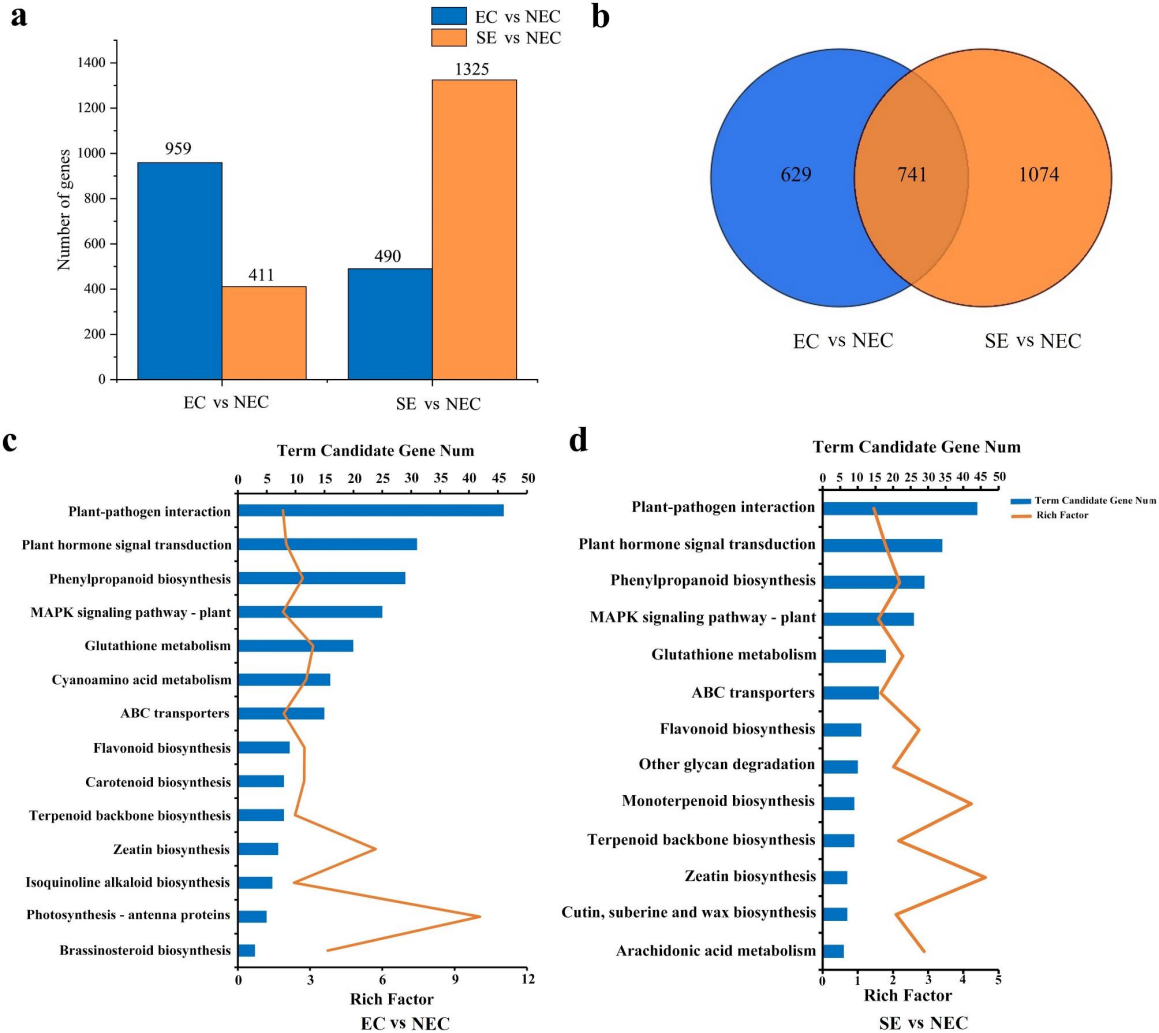
Analysis of differentially expressed genes in embryogenic callus and somatic embryos. **(a)** Comparison of the number of up-regulated and down-regulated differentially expressed genes between the two groups. **(b)** Venn diagram of differentially expressed genes between the two groups. **(c)** KEGG pathway enrichment analysis of differentially expressed genes in non-embryogenic callus and embryonic callus. **(d) **KEGG pathway enrichment analysis of differentially expressed genes in non-embryogenic callus and somatic embryos. EC vs. NEC: embryogenic callus and non-embryonic callus. SE vs. NEC: somatic embryos and non-embryogenic callus. Blue column represents the number of candidate genes for each pathway, yellow broken line indicates the enrichment factors

A total of 188.68 Gb clean data and 58,214 unigenes were obtained by transcriptomic analysis. Significantly DEGs were analyzed according to the thresholds of log_2_ FC ≥ 1, *P-*value < 0.05, and FDR < 0.001, and the FC values of embryogenic callus and non-embryogenic callus (EC vs. NEC), somatic embryo and non-embryogenic callus (SE vs. NEC) were determined by the ratio of the two databases (Table S2). Results showed that 959 unigenes were up-regulated and 411 unigenes were down-regulated in EC vs. NEC (Fig. 3a), and 490 unigenes were up-regulated and 1325 unigenes were down-regulated in SE vs. NEC (Fig. 3a). Venn diagrama of the above three sets of data (Fig. 3b) showed that 741 unigenes overlapped DEGs.

The selected DEGs were further enriched by GO analysis. A total of 21 biological process (BP), 19 cellular component (CC) and 14 molecular function (MF) were enriched in DEGs (Fig. S1). KEGG enrichment analysis of DEGs showed that they were significantly enriched in glutathione metabolism, plant hormone signal transduction, phenylpropanoid biosynthesis and cyanoamino acid metabolism (Fig. S2). This study focused on the KEGG pathway of DEGs between EC vs. NEC and SE vs. NEC. In EC vs. NEC, 31 DEGs were enriched in plant hormone signal transduction, 20 DEGs were enriched in glutathione metabolism, 29 DEGs were enriched in phenylpropanoid biosynthesis, and 16 DEGs were enriched in cyanoamino acid metabolism (Fig. 3c). In SE vs. NEC, 34 DEGs were enriched in plant hormone signal transduction, 18 DEGs were enriched in glutathione metabolism, 29 DEGs were enriched in phenylpropanoid biosynthesis, and 16 DEGs were enriched in ABC transporter. (Fig. 3d). In general, 343 DEGs related to SE were identified based on the analysis of significantly enriched pathways. Among them, 42 DEGs including hormones, methyltransferases, and secondary metabolites were screened for functional annotation and identification (Table S3).

### Analysis of differentially expressed genes related to plant hormone signaling in somatic embryogenesis

**Fig. 4:**
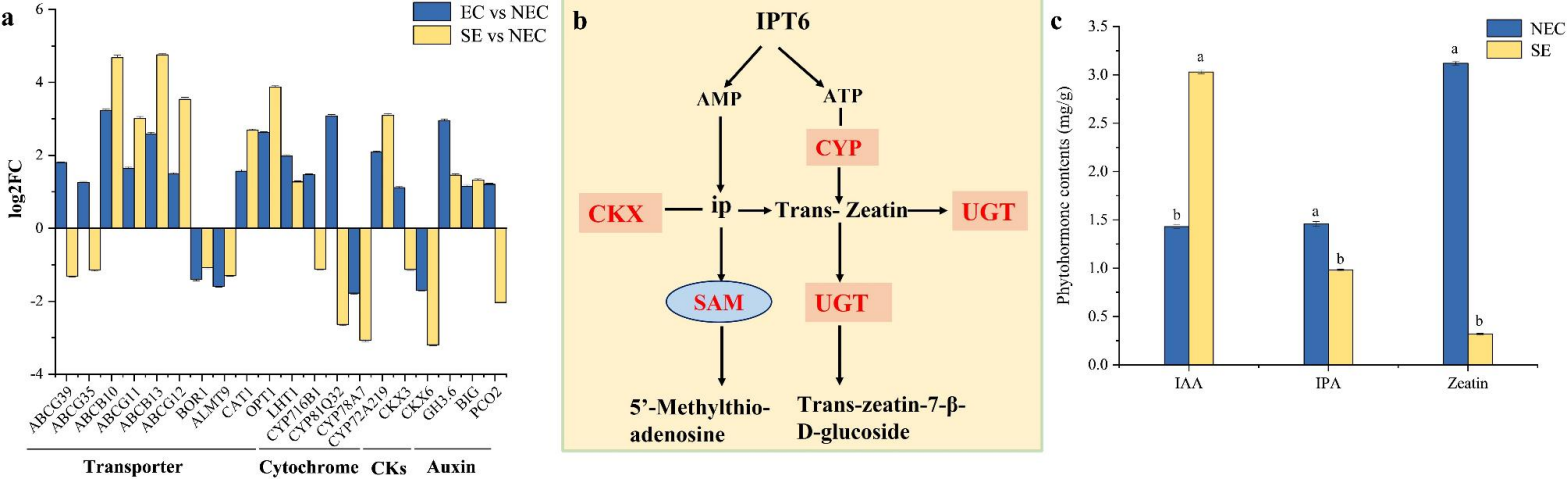
Analysis of plant hormone signal transduction related genes and endogenous hormone content in SE. **(a)** Expression of plant hormone signal transduction related genes. **(b)** Plant hormone signal transduction pathway of differentially expressed genes (www.kegg.jp/kegg/kegg1.html). **(c)** Content of endogenous in SE. NEC: non-embryogenic callus. SE: somatic embryos. EC vs. NEC: embryogenic callus and non-embryonic callus. SE vs. NEC: somatic embryos and non-embryogenic callus. The medium in NEC and SE is MS + 0.5 mg/L 2,4-D + 0.25 mg/L TDZ. Data are the means ± standard error of three replicates. The use of the same lowercase letters indicates that the values were not significantly different according to Tukey’s test (*P* < 0.05). Hormone content in plant samples (ng/g) = detection concentration (ng/ml) × dilution volume (ml) / weight (g)

In terms of plant hormone signal transduction, the DEGs were divided into four categories, including 11 ABC transporter genes, 4 cytochrome P450 family genes, 2 cytokinins, and 3 auxin synthesis related genes (Fig. 4a). Compared to NEC, most ABC transporter family members were up-regulated in EC and SE. CYP450 family genes were up-regulated in EC and down-regulated in SE. Cytokinin dehydrogenase (CKX6) gene was down-regulated in EC and SE, whereas auxin synthesis related genes were up-regulated. Comprehensive analysis of DEGs related to plant hormone signal transduction showed that they were significantly up-regulated during EC, and CYP450 family genes and *CKX6* were down-regulated in SE. *CKX6* is significantly down-regulated in somatic embryogenesis. Indicating that high expression of auxin in ‘Fengdan’ may promote somatic embryogenesis, whereas inhibition of the expression of CYP450 family genes and cytokinins synthesis genes may have a regulatory effect on somatic embryogenesis (Fig. 4b).

The contents of auxin (IAA) and cytokinin (IPA and zeatin) in SE were significantly different among the different treatments (Fig. 4c). However no change was observed in the IP contents (Fig. S3). Compared to NEC, in the SE induced by MS containing 2,4-D and TDZ, the auxin content significantly increased, the cytokinin content significantly decreased. Except for IP, the IPA and zeatin contents were also significantly decreased. The results showed that the IAA content increased and the cytokinin content decreased during somatic embryogenesis, and the IAA content in SE was higher than cytokinin content.

### Differentially expressed genes involved in secondary metabolism

**Fig. 5:**
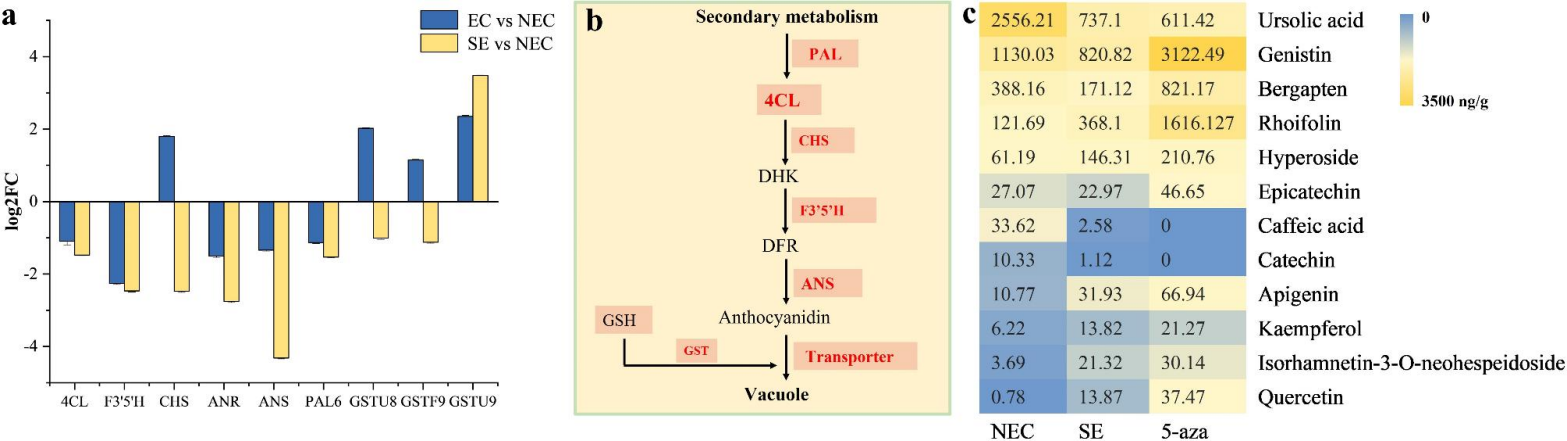
Analysis of secondary metabolism related genes and flavonoid content in SE. **(a)** Expression of secondary metabolism related genes. **(b)** Secondary metabolism pathways of differentially expressed genes (www.kegg.jp/kegg/kegg1.html). **(c)** Content of flavonoids in somatic embryos. NEC: non-embryogenic callus. SE: somatic embryos. 5-aza: somatic embryos induced by 5-azacytidine. EC vs. NEC: embryogenic callus and non-embryonic callus. SE vs. NEC: somatic embryos and non-embryogenic callus. The medium in NEC and SE is MS + 0.5 mg/L 2,4-D + 0.25 mg/L TDZ. The medium in 5-aza is MS + 0.5 mg/L 2,4-D + 0.25 mg/L TDZ + 0.6 mg/L 5-aza. Hormone content in plant samples (ng/g) = detection concentration (ng/ml) × dilution volume (ml) / weight (g)

In our study, DEGs were significantly enriched in flavonoid biosynthesis pathway (Fig. 5). A total of 9 DEGs related to flavonoid biosynthesis were screened in somatic embryogenesis, including phenylalanine ammonia lyase (*PAL*), 4-coumaroyl-CoA (*4CL*), flavonoid 3ʹ,5ʹ -hydroxylase (*F3*ʹ*5*ʹ*H*), anthocyanin synthase *(ANS*), anthocyanin reductase (*ANR*), chalcone synthase (*CHS*), and 3 glutathione transferase family members (*GSTU8, GSTF9, and GSTU9*). Most DEGs in this pathway, except for *GSTU9*, were down-regulated in somatic embryogenesis (Fig. 5a), and expression levels of these DEGs significantly decreased during somatic embryogenesis. Secondary metabolites are catalyzed by PAL to produce 4CL, which is modified by CHS and F3ʹ5ʹH to produce anthocyanidin. It indicated that flavonoid biosynthesis pathway was significantly differentially expressed during somatic embryogenesis in ‘Fengdan’, and flavonoid was mainly accumulated in NEC.

According to the results of transcriptomic analysis, it was found that the DEGs were mainly enriched in secondary metabolism as related to flavonoids. In our study, flavonoids in SE were determined, a total of 12 substances detected significantly different (Fig. 5c). The contents of rhoifolin, hyperoside and apigenin in SE were higher than those in NEC, and the contents of ursolic acid, genistin and bergapten were lower. Furthermore, the addition of 5-azacytidine to induce SE increased the contents of genistin, bergapten, rhoifolin and hyperoside higher than that in NEC, indicating rhoifolin and hyperoside may affect somatic embryogenesis.

### Methyltransferase related genes involved in somatic embryogenesis

**Fig. 6:**
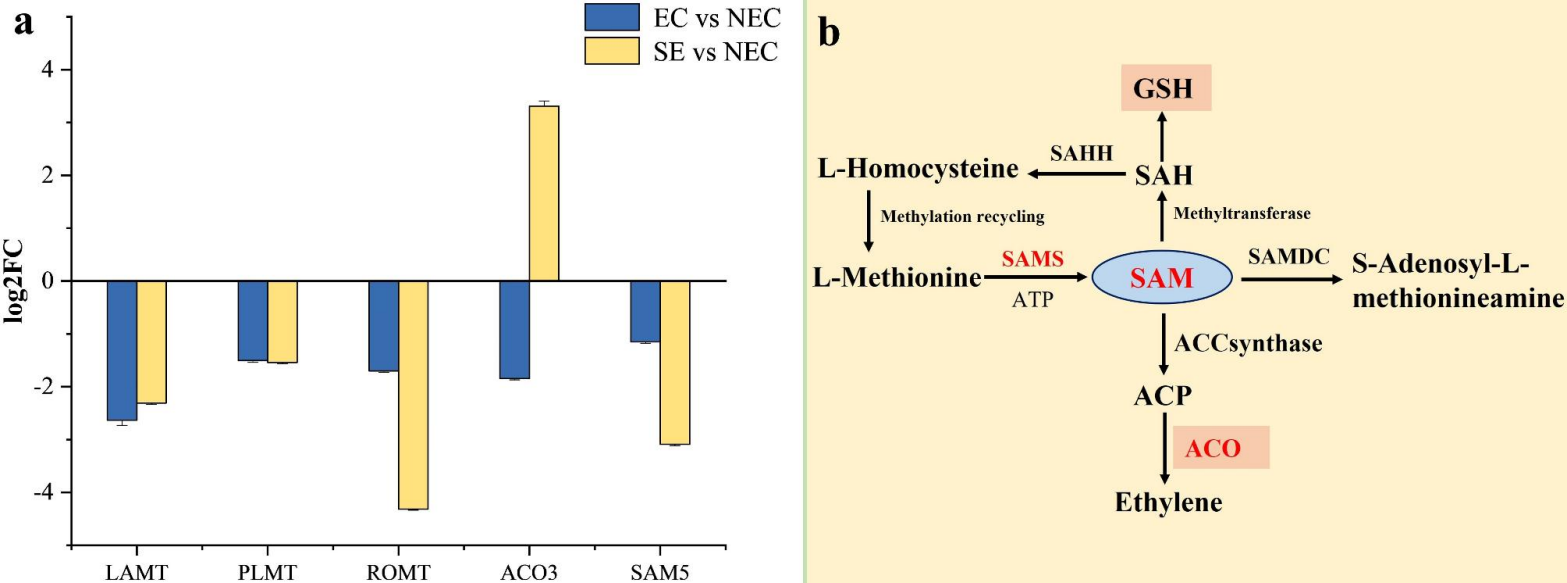
Analysis of methylation-related genes in somatic embryos. **(a)** Expression of methyltransferase-related genes. **(b)** Methyltransferase metabolic pathway of differentially expressed gene (www.kegg.jp/kegg/kegg1.html). EC vs. NEC: embryogenic callus and non-embryonic callus. SE vs. NEC: somatic embryos and non-embryogenic callus

In our study, 5 DEGs of methyltransferase were screened from somatic embryogenesis. They are loganic acid O-methyltransferase (*LAMT*), phosphatidyl-N-methylethanolamine N-methyltransferase (*PLMT*), trans-resveratrol di-O-methyltransferase (*ROMT*), 1-aminocyclopropane-1-carboxylic acid oxidase (*ACO3*) and S-adenosylmethionine synthase 5 (*SAMS5*), and they were significantly down-regulated (Fig. 6a). In addition, *SAM* was catalyzed by *ACO* to produce ethylene (Fig. 6b).

### Transcription factors involved in somatic embryogenesis

**Fig. 7:**
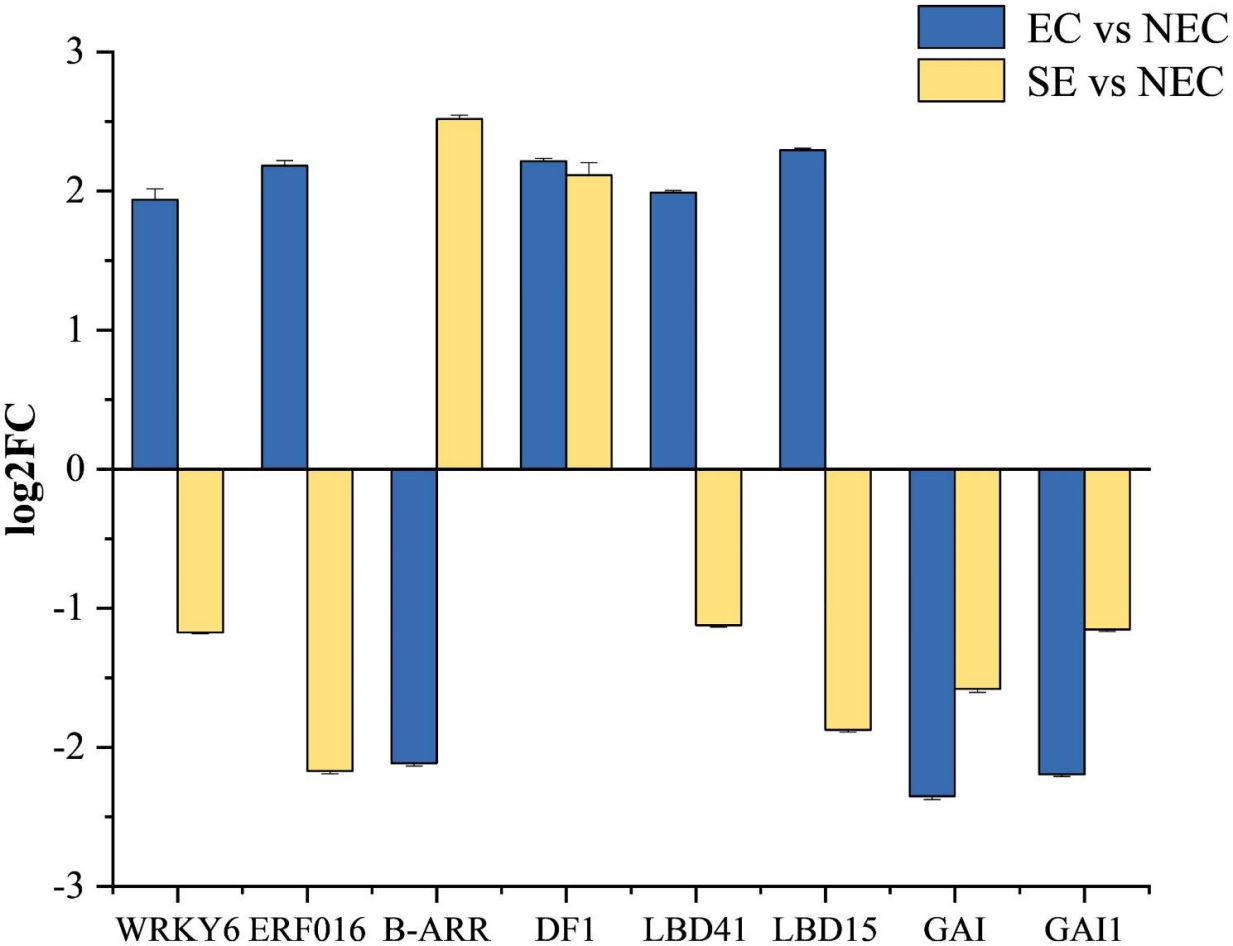
Expression analysis of somatic embryo related transcription factors in ‘Fengdan’. EC vs. NEC: embryogenic callus and non-embryonic callus. SE vs. NEC: somatic embryos and non-embryogenic callus

In this study, 8 differentially expressed transcription factors were screened. Compared with NEC, 5 were up-regulated and 3 were down-regulated in EC, 2 were up-regulated and 6 were down-regulated in SE (Fig. 7). Among them, *WRKY6*, *ERF016* and *B-ARR* transcription factors belong to the *MYB* transcription factor family, other transcription factors were *DF1*, *LBD41*, *LBD15*, *GAI*, and *GAI1*. The results indicate that the occurrence of EC and SE in ‘Fengdan’ was accompanied by expression in transcription factors.

### Verification of differentially expressed genes by qRT-PCR

**Fig. 8:**
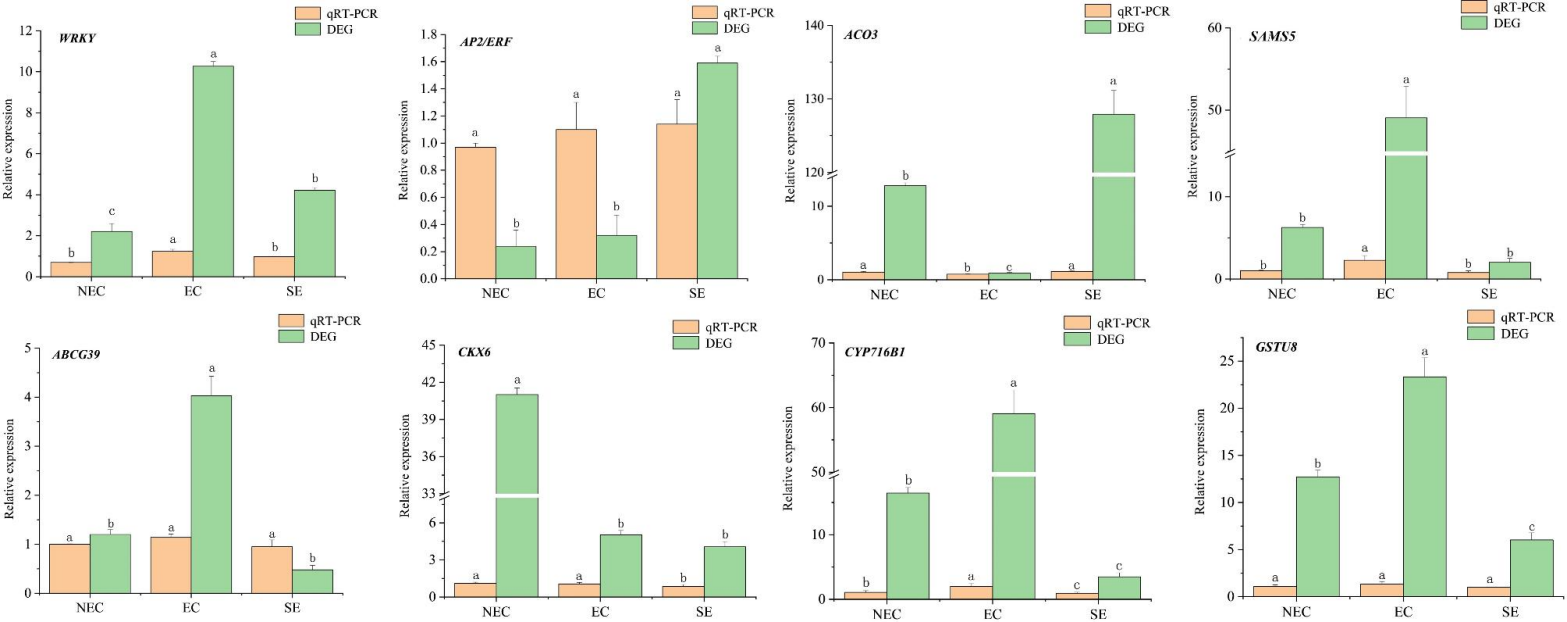
Expression of 8 candidate genes verified by qRT-PCR. Values are means ± SE (n = 3) of three different experiments. Means denoted by letters refer to the significant differences (*P* < 0.05, Duncan’s test)

Eight DEGs associated with somatic embryogenesis were analyzed by qRT-PCR. With *EFl-α* as the internal reference gene. The expression levels were calculated by 2^−ΔΔCT^ method, and the results are shown in Fig. 8. Comprehensive analysis of qRT-PCR and transcriptome sequencing results showed that *ACO3* expression did not change. However, *WRKY*, *ABCG39*, and *CYP716B1* were significantly up-regulated in EC, whereas *AP2/ERF* and *CKX6* were significantly up-regulated in SE. These findings are consistent with the transcriptome sequencing results, indicating the reliability of transcriptome sequencing.

## Discussion

### Effects of different hormone treatments in somatic embryogenesis

De-differentiation is the transition from specialized functional cells to simpler callus without any specialized function, which is one of the important stages of tissue culture [8]. The irregular distribution of auxin in *Coffea canephora* somatic embryogenesis is the main cause of embryo development [25]. Zhou et al. (2017) studied the effects of drought treatment and IAA content on *Pinus massoniana* somatic embryogenesis and observed that 2,4-D induces somatic embryogenesis, and the removal 2,4-D from the induction medium is essential for the late development of SE [26]. Zhang et al. (2021) reported that the use of 2,4-D alone stimulates the production of globular embryos, while the combination with TDZ severely hinders the formation of embryonic cells in *Camellia oleifera* Abel [27].

The induction of EC and SE in ‘Fengdan’ tissue culture is an important problem to be overcome in current research. In our study, EC and SE were successfully induced from hypocotyls and cotyledons of ‘Fengdan’ using the phytohormones 2,4-D and TDZ, indicating 2,4-D plays an important role in somatic embryogenesis. Slice observation of callus showed that vascular tissues formed in the cells when heart-shaped embryos and torpedo embryos were formed. This is consistent with a study that vascular bundles are isolated from maternal tissues during somatic embryogenesis in *Camellia oleifera* Abel [27].

### Effects of plant hormone signal transduction related differentially expressed genes and hormone content in somatic embryogenesis

Auxin signaling is critical for callus induction, involves ARF7, ARF19, and the auxin response factor LBD [28, 29]. Zhang et al. (2019) found that DEGs related to GA and ABA transduction play roles in maize immature embryo regeneration, and promote callus regeneration by regulating the ratio of auxin and cytokinin [30]. Li et al. (2014) observed that the receptor kinase SERK can affect *Picea balfouriana* SE [31]. In addition, ethylene and ACC affect the early differentiation of *Medicago sativa* SE and the addition of amino hydroxyglycine significantly reduces ethylene and ACC levels in SE [32]. It shows that with embryogenesis, the induction of ethylene biosynthesis has a certain effect on embryogenesis. *DICYP* is involved in the phenylpropanoid metabolic pathway in *Dimocarpus longan* SE during the hormone response, which is mainly regulated by miR413. The downstream biosynthesis genes and the isoamyl transferase of *CYP45081E* are significantly up-regulated in yellow callus at different growth period in *Pueraria candollei*, indicating the culture period has a significant effect on the chemical characteristics and biological activities of *Pueraria candollei* callus [33, 34]. Song et al. (2020) demonstrated that somatic embryogenesis may involve water stress and the development of *Lilium pumilum* SE under an appropriate conditions of auxin and cytokinin ratio [35]. Among them, the intracellular auxin level is the determinant of somatic embryogenesis, whereas ABCB and *PILS7* are involved in plant water stress by regulating auxin transport. AtABCG14 is the first discovered cytokinin long-distance transporter that promotes plant growth and development, and cytokinin dehydrogenase overexpression can decrease the content of endogenous cytokinin [36]. This study shows that the expression of ABCB and ABCG transporter family members and the auxin synthesis genes *GH3.6*, *BIG*, and *PCO2* were up-regulated in EC and SE. Cytokinins are generated by cytochrome catalysis and their related genes. CYP450 family genes and *CKX6* were down-regulated in SE, which is consistent with the results of *Lilium pumilum* and maize [30, 35]. Therefore, ABCB and ABCG may regulate somatic embryogenesis by participating in auxin and cytokinin signal transduction during somatic embryogenesis in ‘Fengdan’. Furthermore, exogenous 2,4-D may affect the expression of signal transduction-related genes, providing conditions for callus to enter somatic embryo differentiation.

It is well established that auxin and cytokinin affect somatic embryogenesis, but compared to exogenous factors, endogenous auxin and cytokinin are more important, which directly determine the genesis of somatic embryos [37]. Transcriptomic analysis revealed that auxin and cytokinin synthesis related genes affect somatic embryogenesis. Therefore, the contents of auxin (IAA) and cytokinin (IPA, IP and zeatin) in SE were determined, and the results showed that the auxin content was higher than the cytokinin during somatic embryogenesis. Auxin and cytokinin are essential for de-differentiation and re-differentiation in tissue culture. Cytokinin, an adenine-derived signaling molecule, plays a decisive role in somatic embryogenesis [38]. Endogenous auxin and cytokinin are the main factors determining the responses of cells to exogenous growth regulators in the early stage of somatic embryogenesis in cotton [39]. Somatic embryogenesis in *Arabidopsis* showed that auxin and cytokinin responses are crucial for the formation of radicle meristem from SE [40]. Ohashi-Ito et al. (2014) reported that the transcription factor *LHW-T5L1* targets locally induced *LOG3* and *LOG4*, which triggers interaction between auxin and cytokinin [41]. The findings of our study are consistent with the fact that the auxin content is higher than the cytokinin content, which corresponds to the up-regulated expression of auxin-related genes and the down-regulated expression of cytokinin-related genes in the transcriptomic analysis results. Therefore, the results of hormone determination in this study showed that auxin and cytokinin work together in somatic embryogenesis.

### Effects of secondary metabolite related differentially expressed genes and flavonoid content in somatic embryogenesis

In plant cells, flavonoids are synthesized at different sites in the cytoplasm and then transferred to vacuoles for intracellular transmembrane or long-distance transport with the help of various transporters on the biofilm. Glutathione transferase (GST) can transport the non-enzymatic carrier that catalyzes the binding of anthocyanin-glutathione in the cell and transport it to the vacuole [42]. Stasolla et al. (2010) observed that changes in the redox state of glutathione are key metabolic switches that triggering embryonic growth, and redox glutathione can alter abscisic acid and ethylene synthesis [43]. In the analysis of DEGs, flavonoid-related genes were downregulated in EC and SE. *GST* related genes were upregulated in EC and downregulated in SE. In summary, during somatic embryogenesis in ‘Fengdan’, the upregulated expression of *GST* related genes may promote the binding of glutathione to anthocyanins, thereby regulating somatic embryogenesis.

Peony is a polysaccharide polyphenol plant. More than 8000 phenolic compounds have been identified so far, of which flavonoids account for 60% of phenolic compounds [44]. Current reports on peony tissue culture revealed that phenolic compounds are the main cause of explant browning [45]. However, the effect and molecular mechanisms of flavonoids in somatic embryogenesis has not been studied. In transcriptomic analysis, this study determined the content of flavonoids in somatic embryos, and found that rhoifolin and hyperoside were higher in SE. Hyperoside is a flavonol glycosides compounds, F3ʹ5ʹH is a flavonol biosynthesis related gene, and flavonol biosynthesis pathway related gene expression and hyperoside content have a certain relationship [46]. This study found that the content of hyperoside in SE was higher than that in NEC, whereas *F3*ʹ*5*ʹ*H* was downregulated in SE. This is consistent with the high hyperin content in *Zanthoxylum bungeanum* and the finding of *F3*ʹ*5*ʹ*H* deletion by transcriptome analysis [47]. Therefore, hyperoside may act on *F3*ʹ*5*ʹ*H* to further affect somatic embryogenesis.

### Effect of methyltransferase in somatic embryogenesis

DEGs analysis showed that methyltransferase genes were significantly down-regulated during somatic embryogenesis. As an important methyl donor, SAM exists in both methylation-related pathways and plant hormone-related signaling pathways, and SAM produces ethylene under the catalysis of ACO. This result indicating that SAM may play a regulatory role in somatic embryogenesis of ‘Fengdan’. In addition, since DNA methylation is accompanied by active DNA demethylation, the decrease in the DNA methylation level in embryo culture is not directly caused by the transcriptional activity of genes encoding DNA methylase and demethylase [48]. The key role of DNA methylation in invitro induced morphogenesis compared to plant development has been extensively studied [49, 50]. The SAM-dependent transmethylation pathway is important pathway in de-differentiation, whereas *GhACO3* is involved in ethylene metabolism pathways, and involved in cell division [51]. Munksgaard et al. (1995) demonstrated that the levels of *SAM* and *SAH* increased during somatic embryogenesis, and the level of DNA methylation also increased significantly [52]. We speculate that *SAM* and *SAH* may affect DNA methylation and regulate somatic embryogenesis. Cytokinin dehydrogenase catalyzes the degradation of cytokinin in *Arabidopsis* [53]. Li et al. (2008) reported that the *sahh1-1* can accumulate at higher levels of cytokinin in plants, and transgenic expression changes endogenous cytokinin levels, resulting in changes in the DNA methylation status in s*ahh1-1* conditions [54]. Cytokinin as a S-adenosylhomocysteine hydrolase binding protein, positively regulates methylation pathway genes. A study on DNA methylation in callus differentiation of ‘Fengdan’ showed that the level of DNA hypermethylation was higher during embryogenic callus differentiation [34]. Therefore, we speculate that the downregulated expression of methyltransferase-related genes can be compared with the higher level of DNA methylation in SE.

## Conclusion

**Fig. 9:**
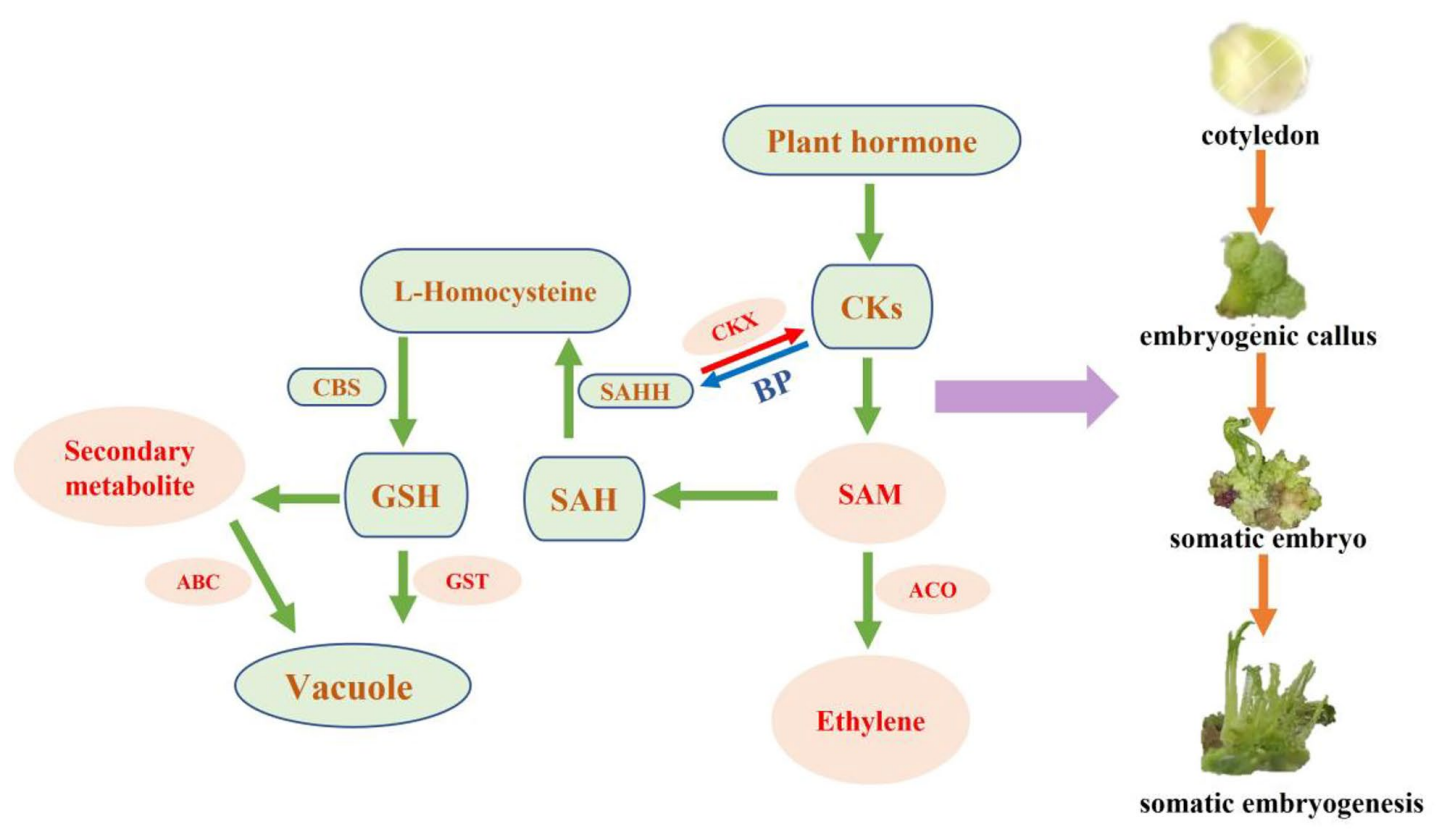
Gene regulation model during somatic embryogenesis in ‘Fengdan’. CKs: Cytokinin, SAM: S-adenosylmethionine. ACO: 1-aminocyclopropane-1-carboxylate oxidase. CKX: Cytokinin dehydrogenase. BP: Binding protein. SAH: S-adenosyl homocysteine. SAHH: S-adenosylhomocysteine hydrolase. CBC: cystathionine synthetase. GSH: Glutathione. GST: Glutathione S-transferase. ABC: ABC transporter protein familys. Red arrows indicate degradation, Blue arrow indicates the binding protein, Green arrows indicate regulation

The results of this study showed that ‘Fengdan’ cotyledons can directly induce somatic embryogenesis. Transcriptomic analysis revealed that auxin and cytokinin may play key roles in somatic embryogenesis. Based on these findings, we propose a molecular regulatory network model of somatic embryogenesis in ‘Fengdan’ (Fig. 9). The down-regulation of *CKX* may lead to the accumulation of endogenous cytokinin, which promotes *SAHH* to positively regulate the genes related to methylation pathway. The up-regulation of *GST* leads to the activation of auxin-related regulatory factors, thereby promoting somatic embryogenesis in callus. Moreover, *SAM* catalyzes homocysteine to produce GSH through transmethylation and cystathionine synthase, leading to EC induction. Downregulation of *SAM* not only caused a decrease in cytokinin synthesis, but also resulted in a decrease in DNA methylation level.

### Electronic supplementary material

Below is the link to the electronic supplementary material.

Supplementary Material 1

Supplementary Material 2

Supplementary Material 3

Supplementary Material 4

Supplementary Material 5

Supplementary Material 6

## Data Availability

The raw sequencing data can be accessed from the NCBI Sequence Read Archive (SRA) platform. (https://submit.ncbi.nlm.nih.gov/subs/sra/) under accession no. PRJNA901227. LC-MS raw data can be accessed from the Figshare platform (https://figshare.com/s/e1f1bbdc5f03fbb7c647).

## Abbreviations

DEG: differential gene
EC: embryogenic callus
FAA: Formaldehyde-acetic acid-ethanol
FC: Fold Change
FDR: false discovery rate
IAA: International Academy of Astronautics
IP: N6-Isopentenyladenine
IPA: Indolepro pionic acid
MS: Murashige and Skoog
MB: vitamin B5 was added to MS medium
MBP: sucrose was replaced with glucose
NEC: non-embryogenic callus
NAA: Indole-3-aceticacid
NCBI: National Center for Biotechnology Information
SE: somatic embryos
SBS: sequencing-by-synthesis
TDZ: thidiazuron
2,4-D: 2,4-dichlorophenoxyacetic acid
6-BA: 6-benzylaminopurine
