# Supplementary material for: Molecular mechanism of somatic embryogenesis in paeonia ostii ‘Fengdan’ based on transcriptome analysis combined histomorphological observation and metabolite determination
Source: BMC Genomics. 2023 Nov 3;24:665. doi: 10.1186/s12864-023-09730-6 (PMC10625268; doi:10.1186/s12864-023-09730-6)
Supplement: Supplementary file 1 — Supplementary Material 1 [file 12864_2023_9730_MOESM1_ESM.docx]

Table S1 qRT-PCR primer information

| Primer name | Sequence F（5’-3’） | Sequence R（5’-3’） |
| --- | --- | --- |
| *PsEF1-α* | TACTCCCTCACAACAACCGC | CTCCTAGCCGTTTCCAGCTC |
| *PsWRKY* | GAGAGGTCTTTGGATGGGCAG | CGGGCGTTGTCACATATTCAAG |
| *PsAP2/ERF* | GACCCGGCTAAGAATGGAGC | CCGGCAATGAACGTTTCGAG |
| *PsSAMS5* | GAATCTGTGAACGAGGGGCA | CACGCAACTTTGCTATCGGG |
| *PsCYP716B1* | GGGCAACAACTATGACGCAC | CTCCCCGGGCATATCCTTTG |
| *PsCKX6* | ATGTCTCTGGTGGCGAGTTG | CCGGCGTTAGACAGAGTACC |
| *PsACO3* | GTCCTTCCGTGGGGACAAAA | ATCGGTGTGGTCACGAAGC |
| *PsGSTU8* | GCCAATCGCAGAGTCTGTTG | ACTTAGCCCAGAAACGTGCC |
| *PsABCG39* | CAAGTCTTTCCACGTTGGGC | TCCATGCTGGAGATGCCATAC |
