# Supplementary material for: Molecular mechanism of somatic embryogenesis in paeonia ostii ‘Fengdan’ based on transcriptome analysis combined histomorphological observation and metabolite determination
Source: BMC Genomics. 2023 Nov 3;24:665. doi: 10.1186/s12864-023-09730-6 (PMC10625268; doi:10.1186/s12864-023-09730-6)
Supplement: Supplementary file 2 — Supplementary Material 2 [file 12864_2023_9730_MOESM2_ESM.docx]

Table S2 Statistical table of differentially expressed genes annotated in callus of ‘Fengdan’

| DEG Set | Total | COG | GO | KEGG | KOG | NR | Pfam | Swiss-Prot | eggNOG |
| --- | --- | --- | --- | --- | --- | --- | --- | --- | --- |
| FD2 vs FD1 | 3459 | 1016 | 2911 | 2401 | 1877 | 3406 | 2469 | 2540 | 2966 |
| FD2 vs FD3 | 8961 | 2455 | 7496 | 6232 | 5106 | 8808 | 6239 | 6413 | 7622 |

Note: DEG Set: Name of differentially expressed gene set. Total: Number of differentially expressed genes annotated. FD2 vs FD1: non-embryogenic callus vs embryogenic callus. FD2 vs FD3: non-embryogenic callus vs somatic embryo.
