# Supplementary material for: Molecular mechanism of somatic embryogenesis in paeonia ostii ‘Fengdan’ based on transcriptome analysis combined histomorphological observation and metabolite determination
Source: BMC Genomics. 2023 Nov 3;24:665. doi: 10.1186/s12864-023-09730-6 (PMC10625268; doi:10.1186/s12864-023-09730-6)
Supplement: Supplementary file 3 — Supplementary Material 3 [file 12864_2023_9730_MOESM3_ESM.docx]

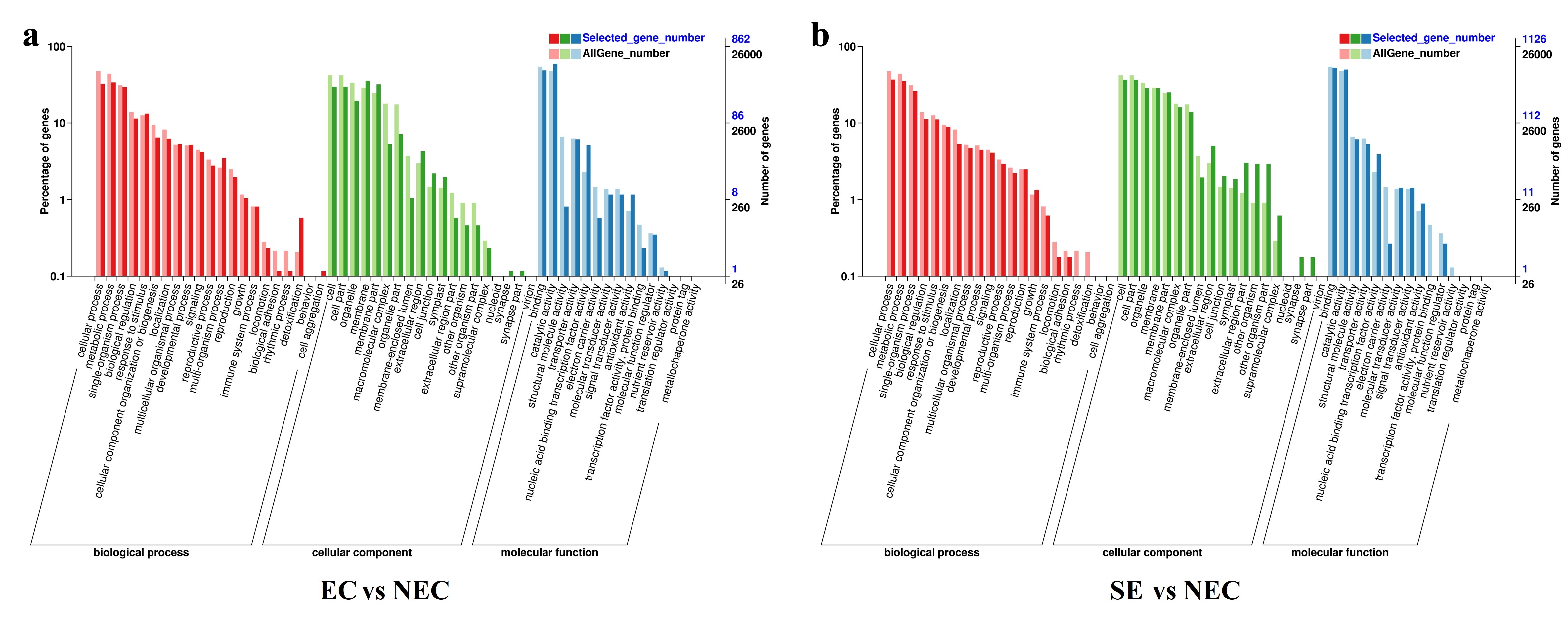


Fig. S1 GO enrichment analysis of differentially expressed genes. (a) GO Enrichment Analysis of Differentially Expressed Genes in EC vs NEC. (b) GO enrichment analysis of differentially expressed genes in SE vs NEC. EC vs NEC: embryonic callus and non-embryogenic callus. SE vs NEC: somatic embryos and non-embryogenic callus.
