# Supplementary material for: Molecular mechanism of somatic embryogenesis in paeonia ostii ‘Fengdan’ based on transcriptome analysis combined histomorphological observation and metabolite determination
Source: BMC Genomics. 2023 Nov 3;24:665. doi: 10.1186/s12864-023-09730-6 (PMC10625268; doi:10.1186/s12864-023-09730-6)
Supplement: Supplementary file 5 — Supplementary Material 5 [file 12864_2023_9730_MOESM5_ESM.docx]

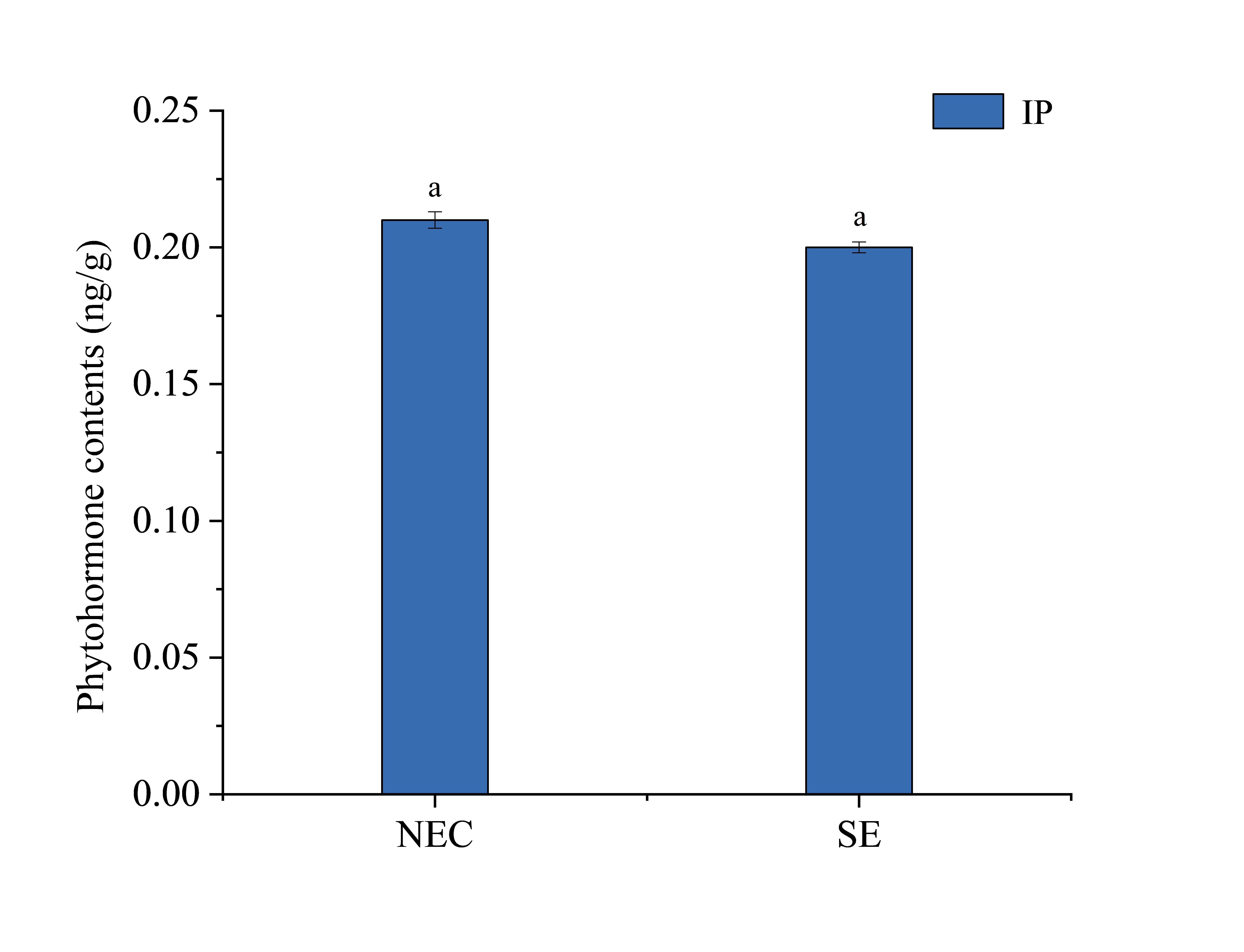


Fig. S3 Content of endogenous hormone in somatic embryos. NEC: non-embryogenic callus. SE: somatic embryos. The medium in NEC and SE is MS+ 0.5mg·L^-1^ 2,4-D + 0.25mg·L^-1^ TDZ. Data are the means ± standard error of three replicates. The use of the same lowercase letters indicates that the values were not significantly different according to Tukey’s test (*P* < 0.05).
