# Supplementary material for: Molecular mechanism of somatic embryogenesis in paeonia ostii ‘Fengdan’ based on transcriptome analysis combined histomorphological observation and metabolite determination
Source: BMC Genomics. 2023 Nov 3;24:665. doi: 10.1186/s12864-023-09730-6 (PMC10625268; doi:10.1186/s12864-023-09730-6)
Supplement: Supplementary file 6 — Supplementary Material 6 [file 12864_2023_9730_MOESM6_ESM.docx]

Table S3 Identification of candidate genes annotated to ‘Fengdan’ somatic embryogenesis .

| Gene ID | Gene Name | Gene Name | Sequence Length (bp) | Homology Species &  Gene Bank Number | CDS Length of Homology Species (bp) |
| --- | --- | --- | --- | --- | --- |
| **Transporter protein** | | | | | |
| psu.G.00011915 | ABCG39 | ABC transporter G family member 39 | 4215 | *Quercus robur*, XM_050387495.1 | 5020 |
| psu.G.00008255 | ABCG35 | ABC transporter G family member 35 | 4281 | *Gossypium raimondii*, XM_012600292.1 | 4991 |
| psu.G.00028145 | ABCB10 | ABC transporter B family member 10 | 1663 | *Rosa chinensis*, XM_024328054.2 | 1311 |
| psu.G.00033169 | ABCG11 | ABC transporter G family member 11 | 1239 | *Vitis riparia*, XM_034855435.1 | 2726 |
| psu.G.00031702 | ABCB13 | ABC transporter B family member 13 | 3525 | *Vitis riparia*, XM_034821137.1 | 4133 |
| psu.G.00023060 | POT5 | Potassium transporter 5 | 3570 | *Juglans regia*, XM_018973605.2 | 2625 |
| psu.G.00006877 | AMT3-3 | Ammonium transporter 3 member 3 | 1338 | *Telopea speciosissima*, XM_043848174.1 | 1538 |
| psu.G.00024623 | ABCG12 | ABC transporter G family member 12 | 1263 | *Quercus robur*, XM_050418590.1 | 2242 |
| psu.G.00030700 | ALMT9 | Aluminum-activated malate transporter 9 | 2187 | *Vitis vinifera*, XM_002272193.3 | 2090 |
| psu.G.00017392 | CAT1 | Cationic amino acid transporter 1 | 1440 | *Camellia sinensis*, XM_028196951.1 | 2226 |
| psu.G.00032704 | LHT1 | Lysine histidine transporter 1 | 965 | *Vitis vinifera*, XM_002265272.4 | 1941 |
| **Cytochrome P450** | | | | | |
| psu.G.00002595 | CYP716B1 | Cytochrome P450 716B1 | 1971 | *Pistacia vera*, XM_031393592.1 | 1585 |
| psu.G.00023445 | CYP81Q32 | Cytochrome P450 81Q32 | 825 | *Vitis vinifera*, XM_002283466.4 | 1976 |
| psu.G.00029098 | CYP714A1 | Cytochrome P450 714A1 | 1594 | *Juglans microcarpa x Juglans regia*, XM_041166269.1 | 1829 |
| psu.G.00028138 | CYP78A7 | Cytochrome P450 78A7 | 1944 | *Nelumbo nucifera*, XM_010247555.2 | 2102 |
| psu.G.00001233 | CYP72A219 | Cytochrome P450 72A219 | 1713 | *Pistacia vera*, XM_031424682.1 | 1850 |
| **Auxin and Cytokinin** | | | | | |
| psu.G.00035246 | GH3.6 | Indole-3-acetic acid-amido synthetase GH3.6 | 1386 | *Herrania umbratical*, XM_021441607.1 | 2273 |
| psu.G.00031051 | BIG | Auxin transport protein BIG | 1167 | *Vitis riparia*, XM_034851073.1 | 15592 |
| psu.G.00030471 | PCO2 | Plant cysteine oxidase 2 | 1196 | *Ricinus communis*, XM_048379945.1 | 1603 |
| psu.G.00006102 | YUCCA10 | Probable indole-3-pyruvate monooxygenase YUCCA10 | 951 | *Hevea brasiliensis*, XM_021804849.1 | 1791 |
| psu.G.00026934 | CKX3 | Cytokinin dehydrogenase 3 | 330 | *Carya illinoinensis*, XM_043121148.1 | 2011 |
| psu.G.00016616 | CKX6 | Cytokinin dehydrogenase 6 | 1218 | *Juglans microcarpa x Juglans regia*, XM_041137605.1 | 2089 |
| **Metabolic pathway** | | | | | |
| psu.G.00027902 | LAMT | Loganic acid O-methyltransferase | 600 | *Citrus clementina*, XM_024182642.1 | 1211 |
| psu.G.00030614 | PLMT | Phosphatidyl-N-methylethanolamine N-methyltransferase | 828 | *Hevea brasiliensis*, XM_021792241.1 | 789 |
| psu.G.00033621 | ROMT | Trans-resveratrol di-O-methyltransferase | 1338 | *Quercus lobata*, XM_031076234.1 | 1281 |
| psu.G.00033565 | ACO3 | 1-aminocyclopropane-1-carboxylate oxidase 3 | 978 | *Paeonia suffruticosa*, DQ337251.2 | 1221 |
| psu.G.00021038 | SAMS5 | S-adenosylmethionine synthase 5 | 1747 | *Vitis riparia*, XM_034835252.1 | 1655 |
| **Secondary metabolism** | | | | | |
| psu.G.00011678 | 4CL | 4-coumarate--CoA ligase 2 | 1110 | *Pistacia vera*, XM_031392722.1 | 2075 |
| psu.G.00024118 | F3’5’H | flavonoid 3',5'-hydroxylase | 1767 | *Paeonia lactiflora*, KM259903.1 | 1509 |

| Table S4. | | | | | |
| --- | --- | --- | --- | --- | --- |
| Gene ID | Gene Name | Gene Name | Sequence Length (bp) | Homology Species &  Gene Bank Number | CDS Length of Homology Species (bp) |
| psu.G.00009935 | CHS | Chalcone synthase | 1044 | *Paeonia suffruticosa*, KJ466964.1 | 1402 |
| psu.G.00027298 | ANR | Anthocyanidin reductase ((2S)-flavan-3-ol-forming) | 1080 | *Carya illinoinensis*, XM_043087336.1 | 1366 |
| psu.G.00029126 | ANS | anthocyanidin synthase | 978 | *Paeonia suffruticosa*, HQ283446.1 | 1269 |
| psu.G.00028533 | PAL6 | Phenylalanine ammonia-lyase 6 | 771 | *Citrus limon*, U43338.1 | 2607 |
| psu.G.00001930 | GSTU8 | Glutathione S-transferase U8 | 651 | *Vitis vinifera*, XM_002275503.4 | 1027 |
| psu.G.00009683 | GSTF9 | Glutathione S-transferase F9 | 1093 | *Ricinus communis*, XM_002530159.4 | 1011 |
| psu.G.00021806 | GSTU9 | Glutathione S-transferase U9 | 940 | *Nicotiana tabacum*, XM_016590882.1 | 902 |
| **Transcription factor** | | | | | |
| psu.G.00002667 | WRKY6 | WRKY transcription factor 6 | 1245 | *Telopea speciosissima*, XM_043870032.1 | 1641 |
| psu.G.00027337 | ERF016 | Ethylene-responsive transcription factor ERF016 | 504 | *Prosopis alba*, XM_028951990.1 | 1012 |
| psu.G.00034608 | B-ARR | Myb family transcription factor IPN2 | 1520 | *Ziziphus jujuba var. Spinosa*,  XM_016034042.3 | 1494 |
| psu.G.00005014 | DF1 | Trihelix transcription factor DF1 | 1752 | *Spinacia oleracea*, XM_023038514.1 | 2433 |
| psu.G.00033941 | LBD41 | LOB domain-containing protein 41 | 756 | *Macadamia integrifolia*, XM_042637917.1 | 1241 |
| psu.G.00027375 | LBD15 | LOB domain-containing protein 15 | 483 | *Manihot esculenta*, XM_021769761.2 | 1581 |
